# Automatically Detecting Pancreatic Cysts in Autosomal Dominant Polycystic Kidney Disease on MRI Using Deep Learning

**DOI:** 10.3390/tomography10070087

**Published:** 2024-07-16

**Authors:** Sophie J. Wang, Zhongxiu Hu, Collin Li, Xinzi He, Chenglin Zhu, Yin Wang, Usama Sattar, Vahid Bazojoo, Hui Yi Ng He, Jon D. Blumenfeld, Martin R. Prince

**Affiliations:** 1 Department of Radiology, Weill Cornell Medicine, New York, NY 10065, USA; sow4006@med.cornell.edu (S.J.W.); zsh4001@med.cornell.edu (Z.H.); col4008@med.cornell.edu (C.L.); xih4004@med.cornell.edu (X.H.); chz4009@med.cornell.edu (C.Z.); yiw4017@med.cornell.edu (Y.W.); uss4002@med.cornell.edu (U.S.); vab4003@med.cornell.edu (V.B.); hfn4001@med.cornell.edu (H.Y.N.H.); 2 The Rogosin Institute, New York, NY 10065, USA; jdblume@nyp.org; 3 Department of Medicine, Weill Cornell Medicine, New York, NY 10065, USA; 4 Department of Radiology, Columbia University Vagelos College of Physicians and Surgeons, New York, NY 10032, USA

**Keywords:** AI, ADPKD, pancreas, pancreatic cyst, imaging biomarker, MRI

## Abstract

Background: Pancreatic cysts in autosomal dominant polycystic kidney disease (ADPKD) correlate with PKD2 mutations, which have a different phenotype than PKD1 mutations. However, pancreatic cysts are commonly overlooked by radiologists. Here, we automate the detection of pancreatic cysts on abdominal MRI in ADPKD. Methods: Eight nnU-Net-based segmentation models with 2D or 3D configuration and various loss functions were trained on positive-only or positive-and-negative datasets, comprising axial and coronal T2-weighted MR images from 254 scans on 146 ADPKD patients with pancreatic cysts labeled independently by two radiologists. Model performance was evaluated on test subjects unseen in training, comprising 40 internal, 40 external, and 23 test–retest reproducibility ADPKD patients. Results: Two radiologists agreed on 52% of cysts labeled on training data, and 33%/25% on internal/external test datasets. The 2D model with a loss of combined dice similarity coefficient and cross-entropy trained with the dataset with both positive and negative cases produced an optimal dice score of 0.7 ± 0.5/0.8 ± 0.4 at the voxel level on internal/external validation and was thus used as the best-performing model. In the test–retest, the optimal model showed superior reproducibility (83% agreement between scan A and B) in segmenting pancreatic cysts compared to six expert observers (77% agreement). In the internal/external validation, the optimal model showed high specificity of 94%/100% but limited sensitivity of 20%/24%. Conclusions: Labeling pancreatic cysts on T2 images of the abdomen in patients with ADPKD is challenging, deep learning can help the automated detection of pancreatic cysts, and further image quality improvement is warranted.

## 1. Introduction

In abdominal imaging studies, pancreatic cysts are important to detect because of their association with pancreatic cancers [1,2,3]. In autosomal dominant polycystic kidney disease (ADPKD), pancreatic cysts are a marker of being 5.9 times more likely to have the PKD2 instead of the PKD1 mutation [4]. PKD2 mutations cause a less aggressive form of the disease, requiring renal replacement therapy later in life, if at all [5]. PKD2 mutations usually do not have seminal megavesicles in males and do not have an established vascular phenotype [6]. Accordingly, it is helpful to identify when pancreatic cysts are present in ADPKD [7].

Pancreatic cysts in ADPKD can be challenging to detect on MRI due to their small size, commonly less than 5 mm. The overwhelming number of cysts in adjacent kidneys and liver also makes pancreatic cysts like needles in a haystack, which requires radiologists to perform a deliberate, meticulous inspection. As a result, pancreatic cysts are commonly missed on abdominal MRI and CT. Deep learning has become promising for lesion segmentation in abdominal imaging [8,9,10,11,12,13,14,15]. Deep learning has been used in segmenting pancreatic cysts on endoscopic ultrasonography, abdominal CT, and MRI [8,9,10,11,12]. To the best of our knowledge, deep learning has yet to be applied in segmenting pancreatic cysts on MRI scans of ADPKD patients, and the presence or absence of pancreatic cysts is rarely included in MRI radiology reports in spite of its importance.

In this paper, we evaluate the ability of an nnU-Net-based deep learning model to automatically detect and segment pancreatic cysts in ADPKD subjects.

## 2. Materials and Methods

This HIPAA-compliant study of existing patient images and medical records was approved by the local institutional review board. The requirement for informed consent was waived. Data can be shared with a data sharing agreement, and the executable model is available at our GitHub repository (https://github.com/Novestars/organ_volume_measurement/tree/pancreatic_cyst) (accessed on 8 July 2024).

### 2.1. Subjects

All subjects met the Pei–Ravine criteria [16] for ADPKD diagnosis and had undergone abdominal–pelvic MRI including T2-weighted sequences with images stored in the Weill Cornell Medicine (WCM) Picture Archiving and Communication System (PACS). Training/validation was performed with T2-weighted images from 146 ADPKD patients, including 76 with pancreatic cysts and 70 without pancreatic cysts. Testing was performed using scans on patients not seen in the training data, including internal (*n* = 40) scanned at WCM, external (*n* = 40) acquired at outside institutions but stored in the WCM PACS for comparison purposes, and test–retest (*n* = 23) ADPKD patients who were scanned twice within a short interval, less than 3 weeks, to assess reproducibility. The first and second scans (scan A and scan B) utilized the same pulse sequences and imaging parameters but not necessarily the same scanner or field strength.

### 2.2. Annotations

Each MRI scan included some combination of axial and coronal T2, axial T2 fat saturation, axial and coronal 3D spoiled gradient echo T1, axial and coronal Steady-State Free Precession, axial Diffusion Weighted Imaging, and, sometimes, gadolinium-enhanced images. However, pancreatic cysts were best visualized on the T2-weighted images, so only axial T2, coronal T2, and axial T2 fat saturation were utilized for model training. MRI pulse sequence details for those sequences are shown in Supplemental Table S1. Pancreatic cysts were labeled by a trained operator (S.J.W.) with knowledge of pancreatic anatomy, and every case was subsequently reviewed by a board-certified radiologist (M.R.P.) with 30 years’ experience in body MRI of ADPKD. Another radiologist with 30 years’ experience (Y.W.) independently labeled all training, internal validation, and external validation cases for cysts. Labeling discrepancies were resolved by consensus. Composed labels for each case summing both radiologists’ contours were generated. Whenever a patient had more than one pulse sequence available, e.g., axial and coronal T2, a cyst identified on one sequence was verified on the other sequence prior to labeling on either sequence. In this way, very small cysts less than 2 mm visible only on a single sequence were excluded, consistent with the original description of pancreatic cysts in ADPKD showing their more frequent occurrence with PKD2 mutations [4]. 

### 2.3. Agreement Evaluation

In order to compare contours created by two expert radiologists for training, internal, and external datasets, composed contours were first created by summing cysts labeled on all contours. Then, each cyst was identified via connectivity, and percent overlap between the composed contour and each observer were calculated. Agreement was defined when the overlap was greater than 50%.

### 2.4. Model Development

Eight models utilizing the standard nnU-Net encoder–decoder architecture [17] were compared for the task of annotating pancreatic cysts in T2-weighted images of ADPKD patients. The network comprised 5 layers with two convolutions per layer. Details regarding patch size, convolution kernel size, stride size, and normalization scheme used for 2D and 3D models are listed in Table 1.

All images used for training and testing were anonymized and converted to NIfTI format. Since the background label dominated these images with few pancreatic cyst labeled voxels, 50% foreground oversampling was used. Weight decay of 3 × 10^−5^, 1000 training epochs, and Z Score normalization were used for all models. Patch size and batch size were adjusted to fit the positive-only dataset or the positive + negative dataset (Table 1). Hyperparameters including loss function, initial learning rate, optimizer, and model configuration (2D [8] or 3D [10,11,12]) were explored to optimize pancreatic cyst segmentation.

### 2.5. Performance Metrics

#### 2.5.1. Internal and External Validation

Ground truth for the 40 internal and 40 external validation cases was determined by consensus of two expert radiologists. The model output for these internal and external cases was then compared to ground truth using the dice similarity coefficient (DSC) at the voxel level and sensitivity and specificity at the scan level using the equations outlined in the Supplementary Materials.

#### 2.5.2. Test–Retest Reproducibility

For 23 ADPKD subjects, scanned twice within a 3-week interval with no intervening clinical events, 6 expert observers (Z.H., C.Z., U.S., Y.W., V.B., and H.Y.N.H.), with experience annotating at least 50 cases each, labeled pancreatic cysts. The pancreatic cysts visible on the T2-weighted images were expected to remain unchanged between the test and retest. Therefore, the test–retest reproducibility of the pancreatic cyst annotation by observer or model was assessed by calculating the percent agreement between scan A and scan B. If both scan A and B classified the subject as positive or negative for the presence of a cyst, agreement was recorded; otherwise, disagreement was recorded. Reproducibility was measured by percent agreement calculated as (# of agreement/# of subjects) × 100.

### 2.6. Outside Radiologist Reports on External Cases

To determine how much value this algorithm could add to the average radiologist, we compared the outside radiologist reports as well as the model output to the gold standard of reference. Reports that did not mention pancreatic cysts were categorized as indicating no pancreatic cyst for the purpose of assessing accuracy.

## 3. Results

**Figure 1:**
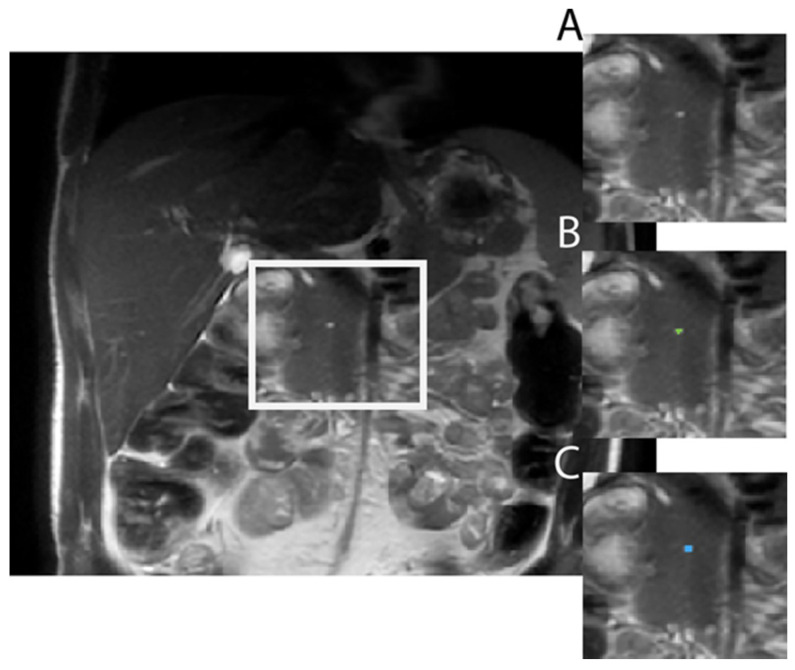
Example of Model output in a 31-year-old male with ADPKD showing (**A**) raw axial T2 SSFSE image, (**B**) the model correctly labeling the pancreatic cyst (green dot), achieving a dice similarity coefficient of 0.62 as compared to (**C**), the ground truth label (blue dot).

Demographic data on the 249 subjects utilized for model training/validation and testing are provided in Table 2. Examples of model output compared to ground truth are shown in Figure 1.

### 3.1. Inter-Observer Variability for Cyst Labeling

Agreement between radiologists for labeling all cysts was limited (52%, 33%, and 25%, respectively, for all images in the training, internal, and external datasets, as shown in Table 3). However, agreement was better for larger cysts, >5 mm, reaching 75% in training data and 47% and 42% in the internal and external test set, respectively.

### 3.2. Model Experiments Results: Internal and External Validation

There were 56 pancreatic cysts in 13 patients on the ground truth evaluation of 40 internal validation cases and 39 pancreatic cysts in 9 patients on the ground truth evaluation of 40 external validation cases. Several combinations of model hyperparameters were explored to create the optimal model for pancreatic cyst segmentation based on internal and external validation with sensitivity and specificity calculated at the scan level (Table 4).

#### 3.2.1. Optimizers

Both stochastic gradient descent (initial learning rate = 0.01) and Adam (initial learning rate = 3 × 10^−4^) were explored. For pancreatic cyst segmentation, Adam consistently converged faster than stochastic gradient descent during training and was therefore used for training.

#### 3.2.2. Loss Functions

The following compound loss (L1, Equation (1)) consisting of the dice similarity coefficient and cross-entropy (*CE*) was used:

(1) $$L1=\frac{TP}{TP+0.5FN+0.5FP}+w_{CE}CE$$

Since this model tends to produce false negatives, cross-entropy weighting ($w_{CE}$) was set at 0.2. A higher cross-entropy weight was also attempted, yet the model performance dropped significantly, and therefore, all models adopted a cross-entropy weighting of 0.2.

To further encourage the model to label cysts, the following second loss function composed of the Tversky Index and cross-entropy (L2, Equation (2)) was also tested:

(2) $$L2=\frac{TP}{TP+\alpha FN+\beta FP}+w_{CE}CE$$

where *α* = 0.1 and *β* = 0.9. Although models trained with *L*2 tend to have higher scan sensitivity, the specificity dropped due to an increased number of false positives.

#### 3.2.3. Datasets with Only Positive Cases and with Positive and Negative Cases

In addition to cases with pancreatic cysts labeled, cases without any pancreatic cysts were included to simulate the clinical frequency of pancreatic cysts appearing in ADPKD and to improve specificity. However, since these negative cases may have exacerbated the class imbalance problem, models trained with positive cases only were also explored.

Models trained on the dataset with both negative and positive cases outperformed models trained on the dataset with only positive cases.

#### 3.2.4. Two-Dimensional vs. Three-Dimensional Configurations

Although 3D segmentation models tend to grasp anatomy better in organ segmentation, 2D models involving less memory may be more effectively trained. We trained models of both 2D and 3D configurations to compare their performances. We found that 2D models performed better on segmenting pancreatic cysts which typically appeared on one slice only.

**Figure 2:**
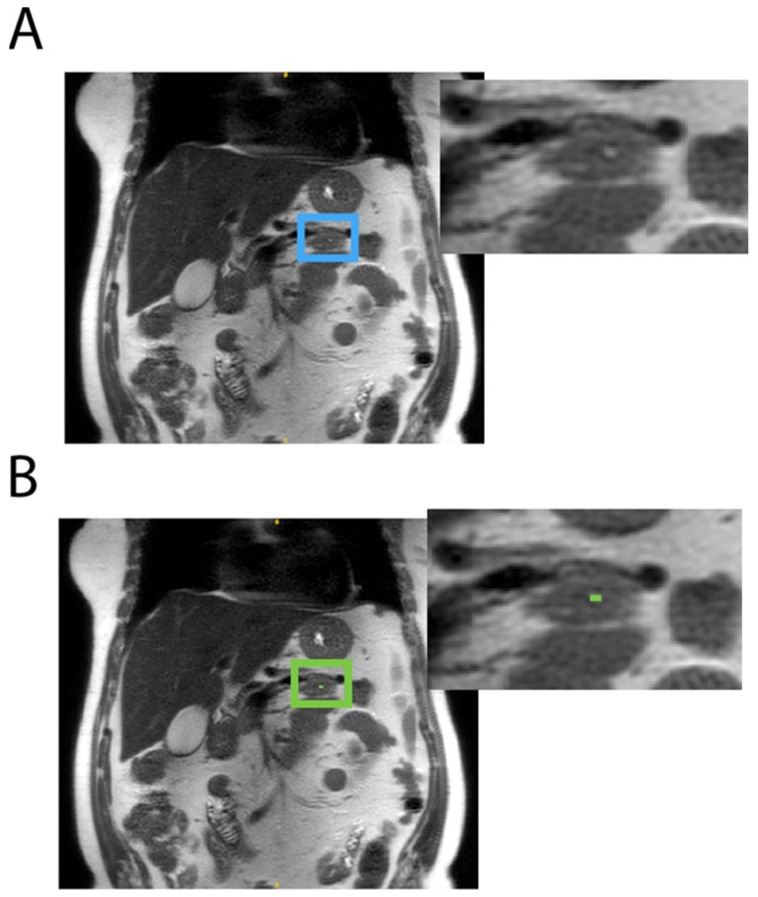
A pancreatic cyst on the coronal T2 SSFSE scan of a 70-year-old female subject was missed by both radiologists (**A**: blue box) which was correctly identified in the model (**B**: green box).

**Figure 3:**
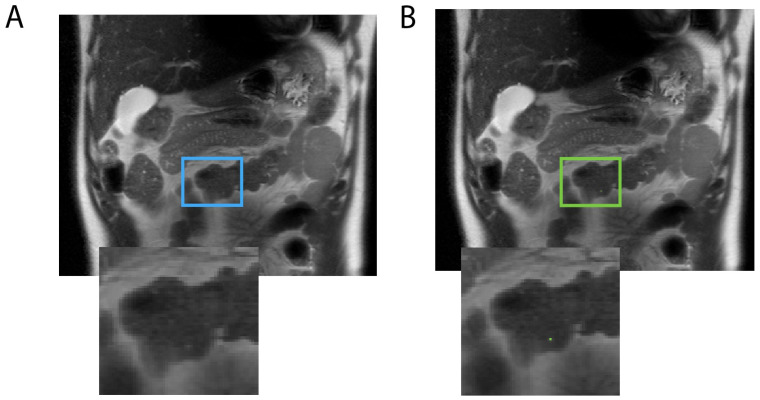
The model mislabeled a bright signal in a 41-year-old male subject’s bowel (**A**: blue box) as a pancreatic cyst (**B**: green box).

Among the eight models trained, the optimal model was the one using a 2D configuration, a compound DSC, and CE loss and was trained on both negative and positive data. DSCs of 0.7 and 0.8 at the voxel level were achieved on internal and external validations, respectively, indicating the accurate identification of pancreatic cysts. Out of 161 scans in the internal and external test sets, only three false positives were found, hence the near-100% specificity: one shown in Figure 2 was due to radiologists missing the pancreatic cyst; the other two were tiny dots labeled on subject’s bowel (Figure 3) and pancreatic duct.

Figure 2 shows a case where expert radiologists failed to label a pancreatic cyst, which was labeled by the model and agreed by both expert radiologists to be correct afterwards. Figure 3 shows examples of model failures.

### 3.3. Test–Retest Reproducibility

Test–retest reproducibility data for 23 ADPKD subjects scanned twice within 3 weeks are shown in Table 5. The optimum model (Table 4 second row) showed better test–retest reproducibility (83%) compared to the mean of six observers (79% on all images—see row 3). Interestingly, observers performed better on axial images compared to coronal images, while the model was indifferent to the imaging plane.

### 3.4. Outside Radiologist Reports on External Cases

Outside radiologist reports were available for 38 of the 40 external validation cases. In these 38 reports that could be examined, outside radiologists mentioned the pancreas in 36 (95%) reports. However, the specific presence or absence of pancreatic cysts was mentioned in only 12 (32%) of those 38 external case reports. Outside reports were also assessed for the evaluation of kidney volume, liver volume, spleen volume, pleural effusions, nerve root cysts, seminal megavesicles, prostate midline cysts, and other imaging features associated with ADPKD (Table S2).

## 4. Discussion

These data from 510 scans in 249 patients demonstrate a deep learning model can detect pancreatic cysts in ADPKD with better reproducibility compared to six expert observers. The model sensitivity was low, consistent with the low agreement between two radiologists and reflecting the limitations of these T2-weighted images. The model specificity was better, providing a promising tool for identifying true pancreatic cysts.

### 4.1. Segmentation Performance

As expected, the model performance was better on cysts with a diameter between 3 mm and 5 mm, which are the types of cysts dominating the training dataset. Most of the errors were false negatives, where the model failed to detect a cyst that was present on both axial and coronal T2-weighted images. It is not surprising that the tortuous pancreatic duct produced false positives, since these structures can appear cyst-like on a single 2D image. Generally, the model was effective at discriminating ducts (common bile duct and pancreatic duct) from pancreatic cysts and made mistakes only with unusually large common bile ducts or tortuous pancreatic ducts.

### 4.2. Comparison to Prior Studies

Early pancreatic cyst segmentation efforts by Zhou et al. in 2017 introduced the two-step segmentation process of first segmenting the pancreas to serve as a mask for limiting the pancreatic cyst search [8]. With 131 contrast-enhanced CT scans, Zhou achieved a mean unsupervised cyst segmentation DSC of 60%. Abel et al. reported 79% sensitivity for detecting pancreatic cysts on contrast-enhanced CT scans using 3D nnU-Net after cropping to slices containing pancreas [10]. The median cyst size was 1.2 cm, and sensitivity dropped to 40% for small cysts less than 50 mm^3^, similar to cysts in our study. Duh et al. reported 93% sensitivity and 80% specificity for detecting pancreatic cysts on contrast-enhanced CT using U-net with skip connections and additive addition gates [11]. However, a true gold standard for pancreatic cysts of cyst aspiration was available for only 6%. Oh et al. evaluated several U-Net model variations applied to cropped endoscopic ultrasound images, finding 98% sensitivity and 99% specificity for the attention U-Net [9]. Endoscopic ultrasonography, however, is invasive, and contrast-enhanced CT involves ionizing radiation plus a contrast agent injection, neither or which are required for MRI. Mazor et al. evaluated pancreatic cyst segmentation on a dataset of 158 MRI studies (not ADPKD) with a training/validation/testing split of 118/17/23, achieving 75% precision and a mean dice score of 0.8 for pancreatic cysts >5mm diameter [12]. This was promising but not suitable for ADPKD subjects whose cysts are mostly 5mm or smaller. Our training data (256 scans from 146 patients and testing data (40 internal, 40 external, and 23 test–retest subjects)) are larger than any of the prior cohorts in the literature and unique in the application to ADPKD subjects who have increased pancreatic cyst prevalence. Furthermore, we evaluated mostly cysts less than 5mm, which were smaller than those in these prior studies. In addition to internal and external validation, our work includes test–retest validation, which was not used in prior studies and shows that our model performance reproducibility is better than human observers. Pancreatic cyst segmentation is substantially more challenging compared to our previous work segmenting liver cysts in ADPKD [15], which are larger and more numerous. While in the pancreas, detecting even a single cyst determines the probability of PKD1 versus PKD2 mutation, nearly all ADPKD subjects have liver cysts; the critical information is total liver cyst volume and liver cyst fraction, which are not substantially affected by missing small cysts.

### 4.3. Clinical Impact

CT and MRI studies on ADPKD patients are complicated to interpret because of the large number of abdomen/pelvis organs affected by this disease, including the kidneys, liver, spleen, pancreas, aorta, IVC, stomach, seminal vesicles, vas deferens, prostate, and nerve roots. For kidneys, meticulous contouring to measure total kidney volume is required, and ideally for the liver as well. Accordingly, it is not surprising that the majority of external radiologist reports have no mention of pancreatic cysts, indicating they were probably not assessed. But with this deep learning model automatically identifying possible pancreatic cysts, this feature is less likely to be missed. The benefits of detecting pancreatic cysts are several. First, the presence of pancreatic cysts, 2 mm or larger, predicts the greater likelihood of the PKD2 over the PKD1 mutation [4]. Second, it identifies cystic lesions that could potentially, albeit rarely, progress to pancreatic malignancies. Third, it saves time for the analysis of ADPKD images and allows the radiologist to focus on other aspects of the case, which is likely happening anyway, since outside radiologists are not even mentioning pancreas cysts in most reports.

### 4.4. Limitations and Future Work

Since our ground truth required cysts to be visible on both axial and coronal images, better performance may be possible with a model that simultaneously trains on both axial and coronal images. Another possibility is to include the pancreas and pancreatic duct mask in the training, although this may not have substantial benefit since the model only produced one false positive for labeling a cyst outside the pancreas and another for labeling the pancreatic duct. Training with larger numbers of patients and prospective validation in a larger cohort will be helpful. Labeling pancreatic cysts on other MRI pulse sequences that allow better visualization of pancreatic ducts, such as magnetic resonance cholangiopancreatography, may also improve model performance and promote agreement between expert radiologists.

## 5. Conclusions

These data from a deep learning model trained/validated on 249 ADPKD subjects show that deep learning can identify pancreatic cysts with high reproducibility and specificity. The currently limited sensitivity will likely improve with more and higher-quality training data.

## Figures and Tables

**Table 1:** Model architecture details.

Positive only Dataset		
Model configuration	2D	3D
Stride size	1 × 1 (layer 1)	1 × 1 × 1 (layer 1)
	2 × 2 (layers 2–5)	1 × 2 × 2 (layers 2–3)
		2 × 2 × 2 (layers 3–5)
Convolution kernel size	3 × 3	1 × 3 × 3 (layers 1–2)
		3 × 3 × 3 (layers 3–5)
Batch size	32	2
Normalization scheme	Z Score	Z Score
Patch size	320 × 320	48 × 192 × 192
**Positive + negative dataset**		
Model configuration	2D	3D
Stride size	1 × 1 (layer 1)	1 × 1 × 1 (layer 1)
	2 × 2 (layers 2–5)	1 × 2 × 2 (layers 2–3)
		2 × 2 × 2 (layers 3–5)
Convolution kernel size	3 × 3	1 × 3 × 3 (layers 1–2)
		3 × 3 × 3 (layers 3–5)
Batch size	26	2
Normalization scheme	Z Score	Z Score
Patch size	320 × 384	48 × 192 × 224

**Table 2:** Demographic data on training/validation and test subjects.

Demographics	Training/Validation		Testing		Total
		Internal	External	Test–Retest	
Patients	146	40	40	23	249
Scans	254	80	81	95	510
DICOM images	15,487	5691	3331	4088	28,597
Male/female (%male)	71:75 (49%)	19:21 (48%)	21:19 (52%)	10:13 (43%)	121:128 (49%)
Age	49 [39, 63]	44 [35, 59]	47 [37, 60]	53 [40, 74]	49 [38, 63]
eGFR ^I^ (mL/min/1.73 m^2^)	60 [38, 84]	63 [41, 88]	76 [51, 90]	62 [42, 132]	61 [39, 85]
BMI (kg/m^2^)	26 [23, 29]	26 [23, 29]	26 [22, 28]	25 [22, 47]	26 [23, 29]
Ht-TKV (mL/m)	766 [398, 1403]	931 [470, 1382]	590 [336, 1027]	595 [366, 2967]	763 [380, 1310]
# Number of patients (%) with pancreatic cysts	63 (43%)	13 (25%)	9 (23%)	15 (61%)	100 (40%)
Total # of pancreatic cysts	404	56	39	46	545
Cyst diameter ^II^ (mm)	4.1 [3.3, 4.9]	4.2 [3.4, 6.3]	4.3 [3.7, 5.8]	5.1 [4.4, 6.4]	4.2 [3.4, 5.2]
Cyst volume ^II^ (mm^3^)	68 [38, 116]	76 [39, 245]	77 [53, 191]	135 [87, 264]	76 [40, 142]
Mayo imaging class 1A	15	2	10	5	32
Mayo imaging class 1B	33	9	10	7	59
Mayo imaging class 1C	36	14	9	5	64
Mayo imaging class 1D	20	9	7	3	39
Mayo imaging class 1E	16	4	2	3	25
Mayo imaging class NA ^III^	26	2	2	0	30
Genotype PKD1	50	17	10	11	88
Genotype PKD2	11	9	4	2	26
Genotype other ^IV^	4	1	1	1	7
Genotype inconclusive	8	3	5	2	15
Genotype unknown	74	11	20	8	113

I. Estimated glomerular filtration rate, excluding patients reaching end stage. II. Diameter and volume of an individual cyst. III. Not applicable—excluding atypical ADPKD and patients receiving renal replacement therapies. IV. IFT140, IFT144, and PKHD1.

**Table 3:** Agreement (cyst level) between radiologist A and radiologist B on label training and testing data.

Cysts Labeled	Training			Internal Test			External Test		
	Axial	Coronal	All	Axial	Coronal	All	Axial	Coronal	All
Total Number of Cysts #	290	148	438	32	28	60	13	19	32
# (%) by 1 radiologist	144 (50%)	65 (44%)	209 (48%)	22 (69%)	18 (64%)	32 (67%)	10 (77%)	14 (74%)	24 (75%)
# (%) by 2 radiologists	146 (50%)	83 (56%)	229 (52%)	10 (31%)	10 (36%)	28 (33%)	3 (23%)	5 (26%)	8 (25%)

**Table 4:** Comparison of performance in eight nnU-Net models for pancreatic cyst segmentation using the same internal validation (*n* = 40) and external validation (*n* = 40) test sets for each model. Sensitivity and specificity are calculated at the scan level defined in the Supplementary Materials. DSC: dice similarity coefficient (mean ± standard deviation); CE: cross-entropy; TI: Tversky Index.

Loss	Configuration	Dataset *	Internal Validation (*n* = 40)			External Validation (*n* = 40)		
			DSC	Sensitivity	Specificity	DSC	Sensitivity	Specificity
L1	2D	pos only	0.6 ± 0.5	0.16	0.78	0.7 ± 0.5	0.1	0.86
		pos + neg	**0.7** ± **0.5**	**0.2**	**0.94**	**0.8** ± **0.4**	**0.24**	**1.00**
	3D	pos only	0.6 ± 0.5	0.36	0.87	0.8 ± 0.4	0.05	0.94
		pos + neg	0.3 ± 0.5	0.56	0.43	0.6 ± 0.5	0.47	0.73
L2	2D	pos only	0.4 ± 0.5	0.64	0.52	0.5 ± 0.5	0.59	0.63
		pos + neg	0.6 ± 0.5	0.2	0.85	0.7 ± 0.4	0.41	0.89
	3D	pos only	0.6 ± 0.5	0.6	0.72	0.3 ± 0.4	0.84	0.35
		pos + neg	0.3 ± 0.4	0.8	0.26	0.4 ± 0.5	0.71	0.44

* pos = positive (*n* = 63), neg = negative (*n* = 83), pos + neg = 146 patients. Bolded row highlights the best overall performing model.

**Table 5:** Test–retest reproducibility for detecting pancreatic cysts on successive MRI scans for the model and 6 expert observers.

Reproducibility (%)	Model			Expert		Observer		
		Mean	1	2	3	4 *	5 *	6 *
Axial (*n* = 23)	87	80	91	70	87	87	74	70
Coronal (*n* = 21)	91	66	85	65	50	75	75	70
All (*n* = 23)	83	79	83	70	87	87	74	70

* Medical doctor.

## Data Availability

The data used for model training and validation are available and can be shared with a data sharing agreement on request from the corresponding author. An executable pancreatic cyst segmentation model can be found at our GitHub repository (https://github.com/Novestars/organ_volume_measurement/tree/pancreatic_cyst) (accessed on 8 July 2024).

## Supplementary Materials

The following supporting information can be downloaded at https://www.mdpi.com/article/10.3390/tomography10070087/s1: Table S1: Imaging parameters of T2-weighted MRI scans used for training/validation and testing, acquired from 1T, 1.2T, 1.5T, and 3T scanners manufactured by GE Healthcare (Milwaukee, WI, USA), Hitachi/FUJIFILM (Greenwood, SC, USA), Philips (Latham, NY, USA), and Siemens (Knoxville, TN, USA); Table S2: Radiology reports from outside institutions reporting ADPKD-relevant quantitative parameters.

## Abbreviations

The following abbreviations are used in this manuscript:

ADPKD	Autosomal dominant polycystic kidney disease
PACS	Picture Archiving and Communication System
DICOM	Digital Imaging and Communications in Medicine
NIfTI	Neuroimaging Informatics Technology Initiative
WCM	Weill Cornell Medicine
DSC	Dice similarity coefficient
CE	Cross-entropy
TI	Tversky Index
