# Supplementary material for: Automatically Detecting Pancreatic Cysts in Autosomal Dominant Polycystic Kidney Disease on MRI Using Deep Learning
Source: Tomography. 2024 Jul 16;10(7):1148–58. doi: 10.3390/tomography10070087 (PMC11281294; doi:10.3390/tomography10070087)
Supplement: Supplementary file 1 [file tomography-10-00087-s001.zip › tomography-2997929-supplementary.pdf]

Automatically Detecting Pancreatic Cysts in ADPKD on MRI using Deep Learning
Supplementary Materials

April 19, 2024

Supplemental Methods

Formulas for calculating model performance metrics

The following medical image segmentation evaluation metrics were used for assessing deep learning segmentation performances. True Positive (TP), True Negative (TN), False Positive (FP), and False Negative (FN) were defined at voxel, cyst, and scan level:

- Dice similarity coefficient, DSC = $\frac{2\times\text{TP}}{2\text{TP}+\text{FP}+\text{FN}}$,
- Sensitivity = $\frac{\text{TP}}{\text{TP}+\text{FN}}$,
- Specificity = $\frac{\text{TN}}{\text{TN}+\text{FP}}$,
- Accuracy = $\frac{\text{TP}+\text{TN}}{\text{TP}+\text{TN}+\text{FP}+\text{FN}}$.

At voxel level, the agreement on whether a single voxel should be labeled as pancreatic cyst was used to evaluate TP, TN, FP, and FN. At cyst level, only TP and FN can be determined: for each cyst labeled on the ground truth, a TP cyst is counted when there is any overlap between the labeled cyst and the model output; similarly, if there is no overlap between model output and the cyst identified from the ground truth, a FN cyst counted. Therefore, at cyst level, only sensitivity can be calculated. At scan level, the agreement between the ground truth and the model on whether there was any pancreatic cyst was used to calculate TP, TN, FP, and FN.

Supplemental Tables

Table S1: Imaging Parameters of T2-weighted MRI Scans Used for Training/validation and Testing, Acquired from 1.5T and 3T Scanners Manufactured by GE (Single-Shot Fast Spin Echo), Philips (Single-SHot Turbo Spin Echo), and Siemens (HAlf-fourier Single-shot Turbo spin-Echo), 1T and 1.2T Open MRI Scanners Manufactured by Philips (Single-SHot Turbo Spin Echo) and Hitachi/FUJIFILM.

	Training/Validation		Testing					
			Internal		External		Test-retest	
	Axial T2	Coronal T2	Axial T2	Coronal T2	Axial T2	Coronal T2	Axial T2	Coronal T2
TE (ms)	82 - 180	79 - 184	88 - 139	78 - 121	60 - 181	60 - 161	86 - 181	87 - 184
Field Strength (T)	1.5 - 3	1.5 - 3	1.5 - 3	1.5 - 3	1.2 - 3	1 - 3	1.5 - 3	1.5 - 3
Bandwidth (Hz/px)	122 - 710	81 - 710	122 - 710	244 - 710	98 - 1461	163 - 780	81 - 710	81 - 725
Matrix Size	240 - 1024 × 256 - 1024	256 - 744 × 250 - 560	240 - 1024 × 320 - 1024	320 - 533 × 320 - 512	192 - 560 × 224 - 640	224 - 768 × 224 - 768	240 - 512 × 256 - 512	256 - 512 × 250 - 512
FOV (cm)	22 - 76 × 22 - 76	34 - 61 × 34 - 50	28 - 84 × 28 - 84	38 - 68 × 38 - 50	26 - 44 × 30 - 44	30 - 44 × 32 - 44	29 - 44 × 30 - 44	38 - 48 × 38 - 48
Slice Thickness (mm)	4 - 8	3 - 8	4 - 6	5 - 6	3 - 7	4 - 8	4 - 10	4 - 10

Table S2: Radiology Reports from Outside Institutions Reporting ADPKD Relevant Quantitative Parameters.

Num. and % External Reports ($n = 38$) Mentioned		
Kidney Length	6	16%
Kidney Length, Width, Height	10	27%
Kidney Length, Width, Height and Volume	9	24%
Kidney Volume Only	9	24%
Largest Renal Cyst Dimensions	15	41%
Liver Craniocaudal Length	3	8%
Liver Volume	0	0%
Splenic Length	2	5%
Spleen Volume	0	0%
Pancreas Volume	0	0%
Pleural Effusion	8	21%
Ascites / Pelvic Fluid	25	66%
Liver Stenosis	5	13%
Anneurysm	24	63%
Umbilical Hernia	2	5%
Nerve Root Cysts	0	0%
Seminal Megavesicles ($n = 19$*)	0	0%
Prostate Midline Cysts ($n = 19$*)	0	0%

* Number of male patients.
